# Characterization of Potential Probiotic Activity of Lactic Acid Bacteria Isolated from Camel Colostrum by Biochemical and Molecular Methods

**DOI:** 10.1155/2023/8334152

**Published:** 2023-10-07

**Authors:** Enas Safi, Moawiya Haddad, Maen Hasan, Sati Y. Al-Dalain, Charalampos Proestos, Shahida A. Siddiqui

**Affiliations:** ^1^Department of Biotechnology, Faculty of Agricultural Technology, Al-Balqa Applied University, Al-Salt, Jordan; ^2^Department of Nutrition and Food Processing, Faculty of Agricultural Technology, Al-Balqa Applied University, P.O. Box 206, Al-Salt 19117, Jordan; ^3^Department of Medical Support, Al-Karak University College, Al-Balqa Applied University, Salt, Jordan; ^4^Laboratory of Food Chemistry, Department of Chemistry, National and Kapodistrian University of Athens, Zografou, Athens 15771, Greece; ^5^Technical University of Munich, Campus Straubing for Biotechnology and Sustainability, Essigberg 3, Straubing 94315, Germany; ^6^German Institute of Food Technologies (DIL e.V.), Prof.-von-Klitzing Str. 7, D-Quakenbrück 49610, Germany

## Abstract

A total of 60 isolates of lactic acid bacteria (LAB) were isolated from Jordanian camel colostrum using biochemical and molecular methods. Two dominant species were identified, and they were *Lactobacillus salivarius* and *Enterococcus faecium*. The entire 60 isolated LAB were tested for their acidity and bile tolerance, antimicrobial activity, and antibiotic sensitivity to test their potential probiotic activity. All 60 isolates were tolerant to different pH concentrations (2, 3, 4, 5, 6, 7, 8, 9, and 10) with different survival rates (%). The entire isolates were also tolerant to different bile salt concentrations (0.2, 0.4, 0.6, 0.8, 1, 2, and 3) with different bile resistance (%). All isolates have a different range of antimicrobial activity against *Staphylococcus aureus*, *E. coli*, and *Salmonella typhimurium*. The 60 isolates were almost sensitive to ampicillin, amoxicillin, and clarithromycin when different concentrations were used except some isolates of intermediate resistance. Only 6% of the isolates were resistant to clarithromycin at a concentration of 15 *µ*g per disc.

## 1. Introduction

Food fermentation using lactic acid bacteria (LAB) is one of the oldest methods of biopreservation. LAB gained attention recently due to their ability to produce antimicrobial substances like bacteriocins and substances used as natural preservatives and to improve the shelf life and/or safety of food. It was proven that probiotic LAB had many positive health effects and it is also safe to be consumed [1].

LAB are known to be a good producer of antimicrobial substances such as hydrogen peroxide, antimicrobial peptides (AMPs), and organic acids, which can be used as food preservatives and they also can be used as an alternative for conventional antibiotics [2].

LAB are identified mainly using phenotypical methods, but nowadays, new rapid, automated, and more sensitive molecular methods are developed to be used as alternative or complementary tools for LAB identification [3]. The identification of LAB using molecular methods is based on the amplification of target sequences of LAB using specific primers, e.g., the amplification of 16S rRNA and 23S rRNA encoding genes, 16S–23S rRNA intergenic region, and ldhD and recA genes [3].

There are many genera of LAB that are used as probiotics for humans and animals, e.g., *Lactobacillus*, *Bifidobacterium*, and *Enterococcus* [4]. Strong evidence proved that many probiotic strains have the ability to resist enteric pathogens using several mechanisms such as food competition, antimicrobial substance production, and stimulation of the immune system. The researchers found that the antimicrobial compounds produced by probiotic LAB could have a protective mechanism against either food or gut pathogens [5].

Camel milk and colostrum are considered an important component of the human diet in many regions of the world [6]. Camel colostrum differs from camel milk in that it has high whey protein content, specifically immunoglobulin (IgG). Both share the main whey components but lack *β*-lactoglobulin [7]. Camel colostrum contains more vitamins, ash, proteins, and minerals than milk. It also contains a significant number of natural antimicrobial agents that enhance the camel's calf immune system [8].

Our study investigated newly isolated strains of lactic acid bacteria from camel colostrum using biochemical and molecular methods. The probiotic properties of the isolated lactic acid bacteria were characterized by biochemical and plating assay methods. To the best of our knowledge, this is the first study to isolate and characterize a probiotic LAB from camel colostrum.

## 2. Materials and Methods

### 2.1. Sample Collection

Six samples of camel colostrum were obtained from multiple sites in Jordan including Shafa Badran, Al-Jafr, Sahab, and Al-Karak. Samples were collected using presterilized 100 ml bottles in an ice box and kept refrigerated until analyzed.

### 2.2. Bacterial References and Pathogens

*Lactobacillus plantarum* ATCC14917 was used as the positive control of probiotic LAB. *Salmonella typhimurium* ATCC14028, *Staphylococcus aureus* ATCC6538, and *Escherichia coli* 8739 were used to test the antimicrobial activity of the LAB isolates of camel colostrum. All bacterial strains were purchased from Microbiologics Inc., USA.

### 2.3. Total Viable Bacterial Count (TVBC), Total Coliform (TC), and LAB Counts

Each colostrum sample was cultivated using the pour plate method on NA, VRB, and MRS agar in triplicate for each of TVBC, TC, and LAB count, respectively. Plates were incubated at 37°C for 48 h. The results were recorded as cfu ml^−1^ [9].

### 2.4. Isolation of LAB

#### 2.4.1. Isolation of LAB Using Biochemical and Culture-Dependent Methods

*(1) Culture-Dependent Method*. Colostrum samples were incubated on a shaker at 100 rpm, at 45°C for 7 days, and then cultivated on MRS agar by the spread plate method. Plates were incubated for 3 days at 37°C using an anaerobic jar (Thermo Scientific AnaeroGen™ 2.5 L sachets (Oxoid™)). Different shapes of the appearing viable bacterial colonies were individually picked from the plates and subcultured again on MRS agar multiple times to purify the isolates. 20% glycerol was added to the pure culture, and they were stored at −20°C until used. Other residual pure cultures were taken to the biochemical tests [10].

*(2) Biochemical Method*. Twenty-four-hour MRS-cys-HCl broth culture from each isolate was tested for catalase production using H_2_O_2_. Isolates which produced bubbles are considered catalase-positive, *S. aureus* was used as positive control, and sterilized broth without inoculation was used as negative control. Catalase-negative colonies are suspected to be probiotic bacteria [11]. Catalase-negative isolates were Gram-stained. Probiotic bacteria colonies are considered Gram-positive [12].

The isolates were inoculated into MRS-cys-HCl broth, and then they were incubated at 10°C and 45°C to test the ability of the isolates to produce CO_2_ as a result of glucose fermentation. *L. plantarum* was used as positive control and sterilized broth was used as negative control. Durham tubes are placed in the media before inoculation to collect the produced gas (Bucio et al., 2006). Phenol red broth was used to test the ability of the isolates to ferment sugars. The broth was prepared by mixing 0.6 g of phenol red with 1.5 L of MRS-cys-HCl broth, and then, it was separated equally into three bottles. Each bottle was mixed with one type of sugar (1%) of each of glucose, lactose, and fructose, and then, they were autoclaved. Each isolate was inoculated into three types of phenol red broth and incubated at 37°C for 12 h. Tubes with the three sugars without inoculation were used as negative control [13]. The change of phenol broth color from red to orange or yellow indicates a positive result.

#### 2.4.2. Isolation of LAB Using the Molecular Method

*(1) DNA Extraction*. Genomic DNA was extracted using the phenol/chloroform method according to Wilson (2001). The precipitate was washed with 70% ethanol, and then, the supernatant was removed, and the pellet was dried and resuspended in 50 *µ*l of TE buffer (Tris-EDTA 1X = molecular grade (pH 8.0) is a buffer composed of 10 mM Tris-HCl containing 1 mM EDTA•Na2).

*(2) Amplification of 16S rRNA Using PCR*. PCR was performed using universal primers (Table 1) to amplify the 16S rRNA according to Melia et al. [14]. All 60 PCR products were purified using Spin Column Purification and sequenced (two-way) (Macrogen, South Korea) using two reactions (forward and reverse) for each one. The consensus sequences were built using Sequence-Alignment Editor (BioEdit version 7.2.5), and then, they were BLAST-searched against the National Center for Biotechnology Information (NCBI) nt database to identify the isolates [15], the results of sequencing were submitted to NCBI, and the accession numbers were obtained.

*(3) Agarose Gel Electrophoresis*. The PCR products were screened by electrophoresis with 1% agarose gel containing 0.05 *μ*l/ml red safe stain. The products were separated for 25 min at 250 volts, and then they were visualized using a UV gel illuminator.

#### 2.4.3. Probiotic Activity

*(1) Antimicrobial Activity*. The antimicrobial activity of the isolated LAB was tested against three pathogens, *Staphylococcus aureus* ATCC6538, *Salmonella typhimurium* ATCC14028, and *E. coli* 8739, using the diffusion method in triplicate. The 60 isolates were cultured for 48 h, and then they were centrifuged for 10 min at 14000 rpm to obtain cell-free supernatant. About 100 *µ*l from each overnight pathogen culture was spread evenly on a Mueller Hinton agar plate, and then a 6 mm well was made using a sterile Pasteur pipette. About 100 *µ*l of each isolate cell-free supernatant was put in the well. The results were recorded as free zone diameter (average for 3 replicates) using a special ruler, after 24 h incubation at 37°C [9, 16].

*(2) Acidity and Bile Salt Tolerance*. MRS-cys-HCl broth was adjusted to different pH and bile salt concentrations. The pH of the MRS broth was 2, 3, 4, 5, 6, 7, 8, 9, and 10, while the bile salt concentrations were 0.2, 0.4, 0.6, 0.8, 1, 2, and 3%. MRS broth without the addition of bile salt and another one at normal pH (6.7) were used as a negative control. The OD of the 60 isolates and the positive control (*L. plantarum*) were adjusted to 0.2. About 200 *µ*l from each isolate was inoculated into 1800 *µ*l of different pH and bile salt MRS broth concentrations in triplicate. The cultures were incubated at 37°C for 24 h, and then the OD was measured at 600 nm [17]. For acidity tolerance, any increase in the OD value after incubation time indicates that the isolate is resistant to acidity, and the survival rate was calculated for each isolate according to the following equation:

(1) $$\begin{array}{c} \text{Survival rate}\left(\%\right)=\left[\frac{\text{OD}24}{\text{OD}0}\right]*100\%, \end{array}$$

where OD24 and OD0 are the optical density before and after incubation, respectively [18].

For bile salt tolerance, the isolate was considered tolerant if there was a 0.3 unit change in its OD value after inoculation, whereas bile salt resistance (%) was calculated for each isolate as follows [19]:

(2) $$\begin{array}{c} \text{Bile salt resistance}\left(\%\right)=\left[\frac{\left(\text{OD}600\text{ in MRS broth with bile salt}\right)}{\left(\text{OD}600\text{ in MRS broth without bile salt}\right)}\right]*100\%. \end{array}$$

*(3) Antibiotic Sensitivity Test*. Isolated LAB were tested for their susceptibility to clarithromycin, ampicillin, and amoxicillin antibiotics using the overlay disc diffusion method in triplicate. The concentrations of antibiotics were as follows: 10 and 30 *µ*g disc^−1^ for ampicillin, 10 and 30 *µ*g disc^−1^ for amoxicillin, and 15 and 30 *µ*g disc^−1^ for clarithromycin. The OD of all the 60 isolates and control (*Lactobacillus plantarum*) cultures were adjusted to 0.2 (108 CFU ml^−1^). MRS agar plates were overlaid with 1% soft agar which contained about 200 *µ*l of each culture, then paper discs were placed on the plates and antibiotics were applied onto the discs at different concentrations, and the diameters of inhibition zones were measured after 24 h incubation at 39°C using a special ruler [19]. The average of the three replicates was recorded for each treatment, and then the antibiotic sensitivity was determined using Clinical and Laboratory Standard Procedures (Papich [20]) for clarithromycin [21] and Clinical and Laboratory Standard Procedures (CLSI M31-A3, [22]) for ampicillin and amoxicillin [23].

#### 2.4.4. Statistical Analysis of Data

The data from the experiments were statistically analyzed using the SAS package system version 9.4. The data were subjected to analysis of variance (ANOVA), and *P* values less than 0.05 were considered significant for the analysis. Differences between interval times of treatment means were determined by the least significant difference (LSD) test at 5% confidence interval.

## 3. Results

### 3.1. Bacterial Counts

#### 3.1.1. Total Viable Bacterial Count (TVBC) and Total Coliforms (TC)

The log10 cfu ml^−1^ of colostrum samples was analyzed for TVBC and TC. The log10 average of TVBC counts was between 7.9 and 8.1. No coliform colonies were observed for all 6 camel colostrum samples.

#### 3.1.2. LAB Count

The log10 cfu ml^−1^ of colostrum samples was analyzed in LAB. The log10 average of LAB count was between 7.3 and 7.5 in anaerobic conditions for all 6 colostrum samples.

### 3.2. Isolation of LAB

#### 3.2.1. Biochemical and Culture-Dependent Methods

A total of 60 isolates of LAB with different morphologies were isolated from 6 samples of camel colostrum collected from the south and middle of Jordan; all of them were catalase-negative, were Gram-positive, and have the ability to ferment all tested sugars (glucose, lactose, and fructose). In the CO_2_ production test, no gas bubble was noticed in the culture tubes during the incubation period of the isolate broth at both 45°C and 10°C.

#### 3.2.2. Molecular Method

**Figure 1:**
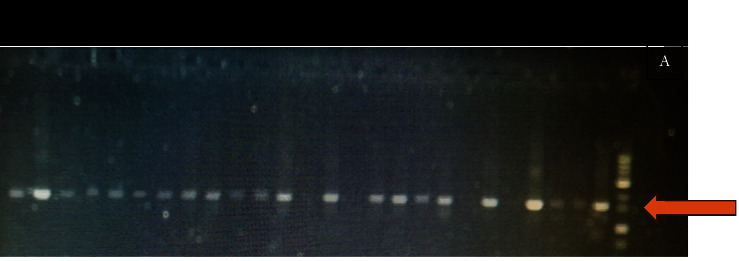
Agarose gel electrophoresis 1% for LAB 16SrRNA gene; the size of amplicons is around 1500 bp compared with the DNA ladder at the arrow pointing in the picture. Lane L: 1 kb DNA ladder; Lane +ve: positive control (*L. plantarum*); Lanes 1–25 represent isolates number.

The PCR results were the same among all isolates, and the amplicon size was about 1500 bp compared to the DNA ladder (Figure 1). Some consensus sequences were analyzed using BLASTn against the NCBI nt database, and the potential identification for each isolate was obtained. Isolates nos. 1.6, 1.7, 2.6, 3.15, 4.1, and 5.5 were identified depending on forward BLAST results, while isolates nos. 4.3 and 6.6 were identified depending on the reverse blast results, and two dominant species were identified. They were *Lactobacillus salivarius* and *Enterococcus faecium* as shown in Table 2. The accession numbers for the 60 isolates sequences were obtained and are recorded in Table 3.

### 3.3. Screening of Probiotic LAB

#### 3.3.1. Antimicrobial Test

All 60 isolates showed different ranges of antimicrobial activity. Isolates 1.5, 1.6, 2.7, 3.7, 3.8, 3.15, and 5.10 have the strongest activity against *S. aureus* ATCC6538. Isolates 1.6, 1.10, 2.9, and 3.9 have the highest activity against *E. coli* 8739, and isolates 1.2, 1.3, 1.5, 1.6, 1.10, 3.5, 3.6, 3.7, 3.8, 3.13, 6.2, and 6.3 showed the strongest activity against *S. typhimurium* ATCC14028. Therefore, isolate 1.6 showed the strongest antimicrobial activity against the three above pathogens, and the results are recorded in Table 3.

#### 3.3.2. Acidity and Bile Salt Tolerance

All isolates were tolerant to all pH concentrations with different survival rates. The survival rates (%) were calculated using the average of the triplicate OD values. The lowest survival rates (%) of all the 60 isolates at pH 2, 3, 4, 5, 6, 7, 8, 9,10, and 6.7 (negative control) were for isolate no. 2.9 (79%), and other isolates showed an increased growth in the acidic environment above the survival rate (more than 100%).

All 60 isolated lactic acid bacteria resist bile salt at different concentrations (0.2%, 0.4%, 0.6%, 0.8%, 1%, 2%, and 3%) with different bile resistance (%). Isolate no. 5.9 recorded the maximum bile tolerance (%) against all bile salt concentrations, while isolate no. 3.2 recorded the minimum bile salt resistance (%) against all bile salt concentrations.

#### 3.3.3. Antibiotic Sensitivity Test

The results revealed that 100% of the isolates were sensitive against both ampicillin 30 *µ*g disc^−1^ and amoxicillin 30 *µ*g disc^−1^ antibiotics. Only 4 isolates (6%) were resistant to clarithromycin 15 *µ*g disc^−1^. There were many isolates showing intermediate resistance as follows: twenty-two isolates (36%) against clarithromycin 15 *µ*g disc^−1^, five isolates (8%) against ampicillin 10 *µ*g disc^−1^, four isolates (6%) against amoxicillin 10 *µ*g disc^−1^, and 3 isolates (5%) against clarithromycin 30 *µ*g disc^−1^ (Table 4).

## 4. Discussion

The current study aimed to characterize the potential probiotic activities of LAB isolated from camel colostrum collected from different areas of Jordan including Amman, Al-Jafr, Sahab, and Al-Karak.

In this study, sixty different shape colonies were isolated from camel colostrum. Biochemical tests such as CO_2_ production, catalase production, carbohydrate fermentation, phenotypic test (Gram stain), and molecular-based test (PCR) were performed to screen LAB from the isolated colonies. All the 60 isolates were Gram-positive, catalase-negative, have the ability to ferment different sugars (lactose, glucose, and fructose), and cannot produce CO_2_. This result indicates that all the 60 isolated colonies were homofermentative LAB. These results coped with those of several previous studies [10, 24].

PCR products for the 60 isolates and the positive control (*L. plantarum*) were about 1500 bp. The size was similar to LAB 16S rRNA size which ensures that all of the 60 isolates are LAB and this result is in agreement with previous studies of Bulut [25] and Naeem et al. [26], which identified LAB using the molecular-based technique (PCR) and the amplicons size was 1500 bp, which is similar to the amplicons of this study.

In this study, the identification of 60 isolates was inspected based on the sequencing of the PCR products. The results of the taxonomy revealed that there are two main dominant species found in Jordanian camel colostrum: *Lactobacillus salivarius* and *Enterococcus faecium*.

There are many previous studies about the isolation of LAB including both *Lactobacillus salivarius* and *Enterococcus faecium* from camel milk, which supports the results of this study [27, 28].

In this study, the isolated LAB were screened for potential probiotic activities. The results of acidity tolerance revealed that all the 60 isolates are tolerant to different pH concentrations (2, 3, 4, 5, 6, 7, 8, 9, and 10) as expected for probiotics to have the ability to survive within the varied pH of the intestinal tract, and these results were in agreement with the previous studies which proved that some *Enterococcus* species can survive in the pH range between 2.0 and 9.5 [29]. Other studies proved that *Enterococcus faecium* can survive at low pH (1.5 and 3) with the survival rate % between 43 ± 3.0 and 79 ± 4.5 [30], and according to Sanhueza et al. [31], some strains of *Lactobacillus salivarius* can survive for 24 h at pH 2.6.

The survival rate % results for the 60 isolates under different pH concentrations showed that the minimum survival rate was 78.8 (%) at pH = 2 while the maximum survival rate was 1166.5 (%) at pH = 10.

Probiotic needs to be bile salt tolerant to survive within the intestine. The bile salt resistance (%) results for the 60 isolates showed that all of them could resist bile salt with different bile salt concentrations (0.2, 0.4, 0.6, 0.8, 1, 2, and 3), and these results support that all the isolates are probiotic bacteria. These results are in agreement with previous studies which said that some strains of *Enterococcus faecium* can survive in media with 1% bile salt concentration with a survival rate of up to 98% [32], and other study proved that some strains of *Lactobacillus salivarius* can resist bile salt with 1% concentration [33].

The results of bile salt resistance (%) for the 60 isolates under different bile salt concentrations showed that the minimum bile resistance % was 21.4% against 3% bile salt and the maximum bile salt resistance % was 99.9% against 0.2% bile salt. These results are expected as the higher the bile salt concentration, the lower the bile salt resistance % of the isolate.

Antimicrobial activity is necessary for LAB to be considered as probiotics, and probiotics need the antimicrobial activity to protect its environment either it is intestinal or food from different pathogens [34]. Antimicrobial activity is the most important criterion for probiotics to be used in an industrial scale as a starter culture and/or as a natural preservative for food [35].

In this study, the antimicrobial activities of the 60 isolates were tested against *S. aureus*, *E. coli* 8739, and *S. typhimurium* ATCC14028. The results revealed that all 60 isolates have the ability to resist the three pathogens in different ranges. Isolate no. 1.6 has the strongest antimicrobial activity against the three above pathogens. These results are in agreement with the previous studies of Murry et al. [36] and Li et al. [37], which proved that some species of *Lactobacillus salivarius* can resist the three above pathogens, and the results of this study are supported by a study which said that some strains of *Enterococcus faecium* are known to be a good producer of bacteriocins and they have a strong antimicrobial activity against all of *E coli*, *Staphylococcus aureus*, *and Salmonella typhimurium* [38].

The results of antibiotic sensitivity revealed that all the 60 isolates were sensitive against ampicillin 30 *µ*g disc^−1^ and amoxicillin 30 *µ*g disc^−1^, while 8% of the isolates showed intermediate resistance against ampicillin 10 *µ*g disc^−1^ and 6% against amoxicillin 10 *µ*g disc^−1^. The sensitivity of the isolates against both ampicillin and amoxicillin corresponds with the previous studies [17, 39–41], which proved that many strains of *Lactobacillus* spp., *Enterococcus* spp., *L. salivarius*, and *Enterococcus faecium* are sensitive to the majority of antibiotics, especially to ampicillin and amoxicillin. The results of this study are in disagreement with the previous results which proved that the strains of *Enterococcus faecium* isolated from clinical specimens have ampicillin and amoxicillin resistance activity [42, 43]. Only 6% of the total 60 isolates were resistant to clarithromycin 15 *µ*g disc^−1^. 3 of them were identified as *Enterococcus faecium*, while 36% of the 60 isolates showed intermediate resistance to the same antibiotic concentration. These results are in agreement with the studies which found that macrolide-resistance genes such as ermB and msrA/B were found in some strains of *Enterococcus faecium* [44], and another study revealed that the ermB gene was found in strains of *Enterococcus faecium* and both ermB and msrC genes were found in a strain of *Lactobacillus salivarius* too [45]; these studies may explain the ability of some isolates to resist macrolide (clarithromycin) antibiotic in this study.

The results of the antibiotic sensitivity test were expected since the samples were collected from camels which were reared in wild areas and they did not expose to antibiotics from either food or medicine, and they may not be able to gain antibiotic resistance.

The results of the biochemical and culture-dependent methods of LAB isolation and the results of probiotic activity screening are supported by the results of the molecular analysis method. As in both the methods, the main identified species were *Lactobacillus salivarius* and *Enterococcus faecium*, which are considered usually as probiotic LAB [46, 47].

## 5. Conclusion

LAB were isolated from Jordanian camel colostrum. *Lactobacillus salivarius* and *Enterococcus faecium* are the two dominant species that were identified. All isolates were tolerant to different concentrations of pH and bile salt, and they were antagonists against *S. aureus*, *E.coli*, and *S. typhimurium* and sensitive to ampicillin, amoxicillin, and clarithromycin antibiotics. All of these features ensure that the colostrum isolates have probiotic activities. The strong antimicrobial activity of the isolates indicates that they may produce strong AMPs and/or bacteriocins, and the sensitivity of the isolates against antibiotics indicates that they are safe to use as probiotics.

## Figures and Tables

**Table 1:** Universal primer sequences used for lactic acid bacterial 16S rRNA gene amplification.

Primer	Primer sequences
Reverse-1525	5′-AGAAAGGAGGTGATCCAGCC-3′
Forward-27	5′- GAGTTTGATCCTGGCTAG-3′

**Table 2:** Molecular identification of isolated LAB depending on NCBI BLASTn.

Isolate #	Scientific name	Strain	Identical %
1	*Ligilactobacillus salivarius*	BCRC 14759	90.4
2	*Ligilactobacillus salivarius*	BCRC 14759	99.2
3	*Enterococcus faecium*	IN 3531	99.7
4	*Ligilactobacillus salivarius*	BCRC 14759	98.9
5	*Ligilactobacillus salivarius*	BCRC 14759	99.4
6	*Lactobacillus salivarius*	2527	91.3
7	*Lactobacillus salivarius*	SS 02	85.2
8	*Lactobacillus salivarius*	3062	99.3
9	*Lactobacillus salivarius*	3155	99.6
10	*Ligilactobacillus salivarius*	BCRC 14759	98.9
11	*Lactobacillus salivarius*	1720	99.4
12	*Lactobacillus salivarius*	08-3C04	95.8
13	*Enterococcus faecium*	IN 3531	99.6
14	*Lactobacillus salivarius*	P1	85.8
15	*Ligilactobacillus salivarius*	BCRC 14759	99.5
16	*Lactobacillus salivarius*	3155	89.6
17	*Lactobacillus salivarius*	1720	98.9
18	*Ligilactobacillus salivarius*	BCRC 14759	99.2
19	*Ligilactobacillus salivarius*	LS-1356	94.7
20	*Lactobacillus salivarius*	ZDY159a	98.4
21	*Enterococcus faecium*	IN 3531	98.9
22	*Enterococcus faecium*	HN-N3	98.4
23	*Enterococcus faecium*	VVEswe-R	97.9
24	*Lactobacillus salivarius*	M7	94.3
25	*Enterococcus* sp.	HVul.ww1	98.9
26	*Enterococcus faecium*	IN 3531	98.4
27	*Lactobacillus salivarius*	3340	98.9
28	*Enterococcus faecium*	IN 3531	98.5
29	*Lactobacillus salivarius*	1720	98.9
30	*Uncultured organism clone*	ELU0019-T101-S-NIPCRAMgANa_000121	93.7
31	*Enterococcus faecium*	VVEswe-R	99.4
32	*Lactobacillus salivarius*	2192	99.9
33	*Enterococcus faecium*	VVEswe-R	99.3
34	*Lactobacillus salivarius*	3155	99.3
35	*Enterococcus faecium*	Unknown33	96.4
		CAU7020	99.50
36	*Ligilactobacillus salivarius*	BCRC 14759	99.8
37	*Uncultured organism clone*	ELU0026-T115-S-NIPCRAMgANa_000153	97.1
38	*Enterococcus faecium*	IN 3531	99.5
39	*Enterococcus faecium*	E1	91.1
40	*Enterococcus* sp.	HVul.ww1	99.1
41	*Lactobacillus salivarius*	M7	97.4
42	*Ligilactobacillus salivarius*	BCRC 14759	98.8
43	*Ligilactobacillus salivarius*	BCRC 14759	96.3
44	*Lactobacillus salivarius*	3155	99.9
45	*Ligilactobacillus salivarius*	BCRC 14759	99.2
46	*Enterococcus faecium*	V1836	98.1
47	*Ligilactobacillus salivarius*	BCRC 14759	99.7
48	*Lactobacillus salivarius*	3316	99.2
49	*Lactobacillus salivarius*	1720	99.6
50	*Ligilactobacillus salivarius*	BCRC 14759	99.5
51	*Lactobacillus salivarius*	1720	99.6
52	*Lactobacillus salivarius*	Yang	87.6
53	*Lactobacillus salivarius*	1720	99.6
54	*Enterococcus faecium*	IN 3531	100
55	*Enterococcus faecium*	IN 3531	99.9
56	*Enterococcus faecium*	gp34	99.7
57	*Enterococcus faecium*	M-26	98.0
58	*Enterococcus faecium*	M-26	99.7
59	*Enterococcus* sp.	RL1137	100
60	*Enterococcus faecium*	IN 3531	99.9

**Table 3:** The accession numbers of 60 isolates LAB of camel colostrum and their antimicrobial activity against pathogens (degree of growth inhibition).

Isolate no.	Accession #	*S. aureus*	*E. coli*	*S. typhimurium*
1.1	OK037439	++	++	++
1.2	OK037440	++	++	+++
1.3	OK037441	++	++	+++
1.4	OK037442	++	++	++
1.5	OK037443	+++	++	+++
1.6	OK037444	+++	+++	+++
1.7	OK037445	++	++	++
1.8	OK037446	++	++	++
1.9	OK037447	++	++	++
1.10	OK037448	++	+++	+++
2.1	OK037449	++	++	++
2.2	OK037450	++	++	++
2.3	OK037451	++	++	++
2.4	OK037452	++	++	++
2.5	OK037453	++	++	++
2.6	OK037454	++	++	++
2.7	OK037455	+++	++	++
2.8	OK037456	++	++	++
2.9	OK037457	++	+++	++
2.10	OK037458	++	++	++
3.1	OK037459	++	++	++
3.2	OK037460	++	++	++
3.3	OK037461	++	++	++
3.4	OK037462	++	++	++
3.5	OK037463	++	++	+++
3.6	OK037464	++	++	+++
3.7	OK037465	+++	++	+++
3.8	OK037466	+++	++	+++
3.9	OK037467	++	+++	++
3.10	OK037468	++	++	++
3.11	OK037469	++	++	++
3.12	OK037470	++	++	++
3.13	OK037471	++	++	+++
3.14	OK037472	++	++	++
3.15	OK037473	+++	++	++
3.16	OK037474	++	++	++
4.1	OK037475	++	++	++
4.2	OK037476	++	++	++
4.3	OK037477	++	++	++
4.4	OK037478	++	++	++
4.5	OK037479	++	++	++
4.6	OK037480	++	++	++
4.7	OK037481	++	++	++
5.1	OK037482	++	++	++
5.2	OK037483	++	++	++
5.3	OK037484	++	++	++
5.4	OK037485	++	++	++
5.5	OK037486	++	++	++
5.6	OK037487	++	++	++
5.7	OK037488	++	++	++
5.8	OK037489	++	++	++
5.9	OK037490	++	++	++
5.10	OK037491	+++	++	++
6.1	OK037492	++	++	++
6.2	OK037493	++	++	+++
6.3	OK037494	++	++	+++
6.4	OK037495	++	++	++
6.5	OK037496	++	++	++
6.6	OK037497	++	++	++
6.7	OK037498	++	++	++
*L. plantarum*		++	++	++

^a^The sequences were submitted to NCBI, and accession numbers for each sequence were obtained. ^b^Different scores reflect the different degrees of inhibition representing the average of triplicate. Free zones in mm: less than 5 mm no inhibition (−), free zone between 5 and 10 mm (+), free zone between 11 and 17 mm (++), and free zone more than 17 mm (+++).

**Table 4:** The results of antibiotic sensitivity test of a set of isolates against multiple antibiotics with different concentrations.

Isolate #	Ampicillin 30 *µ*g disc^−1^	Ampicillin 10 *µ*g disc^−1^	Clarithromycin 30 *µ*g disc^−1^	Clarithromycin 15 *µ*g disc^−1^	Amoxicillin 30 *µ*g disc^−1^	Amoxicillin 10 *µ*g disc^−1^
*L. plantarum*	S	S	S	S	S	S
1.1	S	S	S	I	S	S
1.2	S	S	S	I	S	S
1.3	S	S	S	I	S	S
1.4	S	S	S	I	S	S
1.5	S	S	S	I	S	S
1.6	S	S	S	I	S	S
1.7	S	S	I	R	S	S
1.8	S	S	S	S	S	S
1.9	S	S	S	S	S	S
1.10	S	S	S	S	S	S
2.1	S	S	S	S	S	S
2.2	S	I	S	I	S	S
2.3	S	S	S	I	S	S
2.4	S	S	S	S	S	S
2.5	S	S	S	S	S	S
2.6	S	S	S	S	S	S
2.7	S	S	S	I	S	S
2.8	S	I	S	I	S	S
2.9	S	S	S	S	S	S
2.10	S	S	S	S	S	S
3.1	S	S	S	S	S	S
3.2	S	S	S	S	S	S
3.3	S	S	S	I	S	S
3.4	S	S	S	S	S	S
3.5	S	S	S	S	S	S
3.6	S	S	S	I	S	S
3.7	S	S	S	S	S	S
3.8	S	S	S	S	S	S
3.9	S	S	S	S	S	S
3.10	S	I	S	I	S	I
3.11	S	S	S	I	S	S
3.12	S	S	S	S	S	S
3.13	S	S	S	S	S	S
3.14	S	S	S	I	S	S
3.15	S	S	S	S	S	S
3.16	S	S	S	S	S	S
4.1	S	S	S	I	S	S
4.2	S	S	S	S	S	S
4.3	S	S	S	R	S	S
4.4	S	I	S	I	S	I
4.5	S	S	S	S	S	S
4.6	S	S	S	S	S	S
4.7	S	S	S	S	S	S
5.1	S	S	S	I	S	S
5.2	S	S	S	S	S	S
5.3	S	S	S	I	S	S
5.4	S	S	S	S	S	S
5.5	S	S	S	S	S	S
5.6	S	S	S	I	S	S
5.7	S	S	S	S	S	S
5.8	S	S	S	S	S	S
5.9	S	S	S	S	S	S
5.10	S	S	S	S	S	S
6.1	S	S	S	S	S	S
6.2	S	S	S	S	S	S
6.3	S	S	I	R	S	I
6.4	S	S	S	I	S	S
6.5	S	I	S	I	S	S
6.6	S	S	I	R	S	I
6.7	S	S	S	S	S	S

^a^The results were determined from the average of the triplicate OD values according to CLSI (M31-A3 [22]) and Papich [20], and the results were recorded as sensitive (S), resistance (R), and intermediate (I).

## Data Availability

The data used to support the findings of this study are available from the corresponding author upon request.
